# Design and Characterization of an Adjustable Passive Flow Regulator and Application to External CSF Drainage

**DOI:** 10.3390/mi14030675

**Published:** 2023-03-19

**Authors:** Eric Chappel

**Affiliations:** Microsystems Department, Debiotech SA, 1004 Lausanne, Switzerland; e.chappel@debiotech.com

**Keywords:** passive valve, flow control, constant flow regulator, external ventricular drain (EVD), cerebrospinal fluid (CSF)

## Abstract

Passive valves that deliver a constant flow rate regardless of inlet pressure changes have numerous applications in research, industry, and medical fields. The present article describes a passive spring valve that can be adjusted manually to deliver the required flow rate. The valve consists of a movable rod with an engraved microchannel. The fluidic resistance of the device varies together with the inlet pressure to regulate the flow rate. A prototype was made and characterized. Flow-rate adjustment up to +/−30% of the nominal flow rate was shown. A simple numerical model of the fluid flow through the device was made to adapt the design to external ventricular drainage of cerebrospinal fluid (CSF). Some insights about the implementation of this solution are also discussed.

## 1. Introduction

Passive flow regulators or flow-controlled valves deliver a constant flow rate regardless of pressure variations [1]. These compact, reliable, maintainable and energy-free valves are widely used in industry for many applications, including water treatment, process water control, centrifugal pump protection, water-saving, irrigation, etc. [2,3]. These passive valves usually have moving parts, and automatic adjustment of flow to pressure conditions is achieved by changing the dimensions of the fluid path. A variation of pressure moves a flexible element of the device to induce a change in the fluidic path cross-section or length. The flexible element can be a silicon membrane [4,5,6,7,8,9,10,11,12,13], an elastomeric membrane [14,15,16,17,18,19,20,21,22,23], a silicone tube [24], a thin flap [25,26,27,28], or a moving piston [29,30].

Several implantable passive flow-controlled valves have been considered for the drainage of cerebrospinal fluid (CSF) from the brain ventricles toward the peritoneum cavity for the treatment of hydrocephalus [7,13,22,23,24,29,31,32,33]. The single flow-control valve available on the market is the OSVII™ (Integra Life Science, Princeton, NJ, USA). Two models are proposed, covering the following ranges of flow rate: 8 to 17 mL/h, to meet the special needs of normal-pressure hydrocephalus (NPH) patients requiring a lower flow rate, and 18 to 30 mL/h [23]. This three-stage passive flow-regulated valve has a flexible silicone membrane with a narrow orifice surrounding a pin of changing diameter. In the first stage, also called the differential pressure stage, the valve operates as a standard differential pressure valve with low hydraulic resistance. In the flow-regulating stage, the membrane goes down and the pin progressively narrows the orifice to maintain the flow constant. The third stage is reached under high-pressure conditions (above 36 mbar). The membrane moves beyond the pin, and the hydraulic resistance is thus considerably reduced. This technology, introduced in 1987 by Cordis, was included in many shunt evaluation campaigns [1,22,34]. In principle, this flow-regulating shunt should limit the risk of overdrainage associated with differential shunts but, in turn, may induce excessive CSF pressure during cerebrovascular fluctuations in patients with limited pressure–volume compensatory reserve [34]. An alternative to account for individual-to-individual variability in CSF production rate and cerebrovascular fluctuations is the implementation of the specific fluidic profile that should allow better control of intracranial pressure (ICP) [32]. A MEMS-based passive flow-regulating valve has been designed to evaluate this solution [13]. The device consists of a flexible membrane with holes and facing pillars that form valves that close progressively as the pressure increases. The resulting reduction of the gap between the membrane and the pillars leads to an increase in hydrodynamic resistance. Numerical simulations have been performed to obtain the targeted flow profile. MEMS technology offers numerous advantages in terms of manufacturing cost, repeatability, and reliability, but the small dimensions of the fluid pathway make the device sensitive to contamination and prone to clogging [13].

The external ventricular drainage (EVD) of CSF is commonly used in intensive care units in patients with elevated ICP. This condition may be associated with tumors, hemorrhage, aneurysm, meningitis, traumatic brain injury, acute obstructive hydrocephalus, etc. Standard external drainage systems consist of a set of different elements fixed to an intravenous (IV) pole, including catheter and fluidic lines, stopcocks, a burette to monitor the flow rate, access ports (for priming, sampling, purging, etc.), a pressure sensor, a check valve (e.g., duckbill valve), a collecting bag and a means to adjust the position of the burette and level the system with the tragus. The fluidic lines exhibit a small fluidic resistance, thus misuse, the patient’s movements, etc., without clamping of the line can lead to severe overdrainage potentially causing patient death [35]. A flow-control valve is, by design, more robust to misuse and modifications of the fluidic line position. The challenge here is to provide a device compatible with a large range of potential CSF flow rates (which is unknown at the beginning of the treatment). In addition, the weaning procedure at the end of the treatment requires a progressive reduction of the flow through the valve. Finally, because the fluid to be drained is often hemorrhagic, the design of the valve should exhibit a good tolerance to contamination, with ideally a purge mode.

A flow-control valve with adjustable means and a purge mode is therefore desirable for this specific application (EVD). The focus here is on the original design of an adjustable passive valve for insertion into the fluid path of a standard external drainage system. Valve setting is also an important feature of other potential applications, such pain management, where the delivery rate is tailored to patient experience. To date, the only adjustable passive valve is a MEMS microvalve, in which the fluid path is adjusted manually or passively adjusted to deliver a constant flow, regardless of viscosity changes due to temperature variation [36]. However, this device is not suitable for EVD, due to its sensitivity to contamination [13].

**Figure 1:**
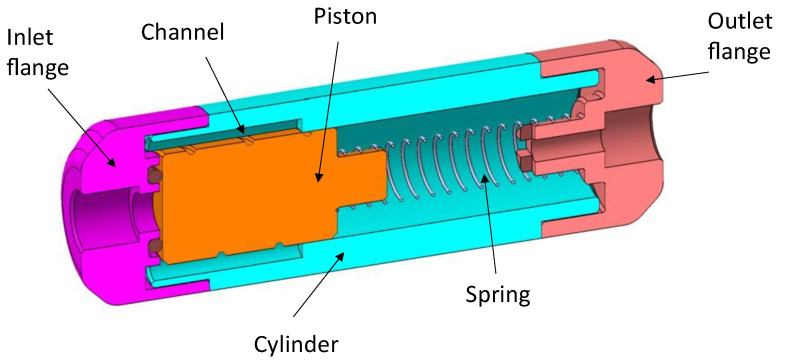
Cross-section of the passive constant flow valve at rest. The piston is engaged in the narrow part of the cylinder. Increasing the pressure leads to a movement of the piston to the right until the pressure force onto the piston is balanced by the spring restoring force. The fluid is forced to flow through the channel engraved into the piston.

The spring valve design that was considered for the internal diversion of CSF from the brain ventricles to the peritoneal cavity [29] was adapted to allow manual adjustment of the flow rate. Indeed, this valve technology offers several competitive advantages that make it an interesting candidate for such a feasibility study. First, the device has relatively large fluid openings that would limit the risk of clogging. In addition, the valve has self-cleaning capabilities and can be easily purged [1,29]. This modular valve is compatible with the standard turning process and therefore compatible with the low cost requirements of a fully disposable EVD system. Finally, adjustability can be obtained in a relatively simple manner as the nonlinearity of the flow profile is obtained with a linear element (the compression spring). Other flow-adjustment features have been theoretically proposed for a MEMS-based valve, but this solution would require a complex and difficult assembly process [33]. The original valve consists of a moving cylinder with a spiral groove and a linear compression spring inside a hollow cylinder with openings (see Figure 1). At rest, the piston is partly engaged into the narrow part of the cylinder. As the pressure force on the piston increases, the piston is engaged further into the cylinder and the fluid is forced to flow through a longer channel length. The force generated by the spring increases linearly with the applied pressure and thus the movement of the piston leads to a linear increase in the channel length as the pressure increases. As the channel cross-section and spiral pitch are constant, the flow is regulated at a predetermined fixed value. At high pressure, the device can either exhibit free flow or an occlusion state.

In the present study, the means to manually adjust the flow rate was analyzed through modeling and experimentation. The fluidic model was presented first followed by a description of the prototypes and the fluidic characterizations. Finally, simulations are shown of an adjustable spring valve design dedicated to EVD.

## 2. Materials and Methods

### 2.1. Modeling

#### 2.1.1. Simplified Model

**Figure 2:**
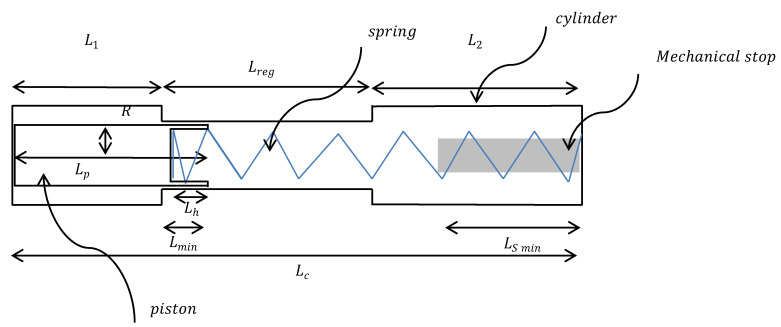
Schematic cross-section of the adjustable flow-control valve. The notations are described in the nomenclature.

The simplified model does not consider the effect of gravity and the singular head losses along the fluid path. The different notations used to model the fluidic behavior of the device are illustrated in Figure 2.

According to [29], the length *L_loop_* of one loop of the helicoidal channel is:

(1) $$L_{loop}=2\pi \sqrt{{\left(R-\frac{D_{h}}{2}\right)}^{2}+b^{2}}$$

where *R* is the piston radius, 2*πb* the helix pitch, and *D_h_* the hydraulic diameter of the channel.

Consequently, the channel length per unit of piston length *α* takes the form:

(2) $$\alpha =\frac{L_{loop}}{2\pi b}=\sqrt{1+{\left(\frac{R-\frac{D_{h}}{2}}{b}\right)}^{2}}$$

**Figure 3:**
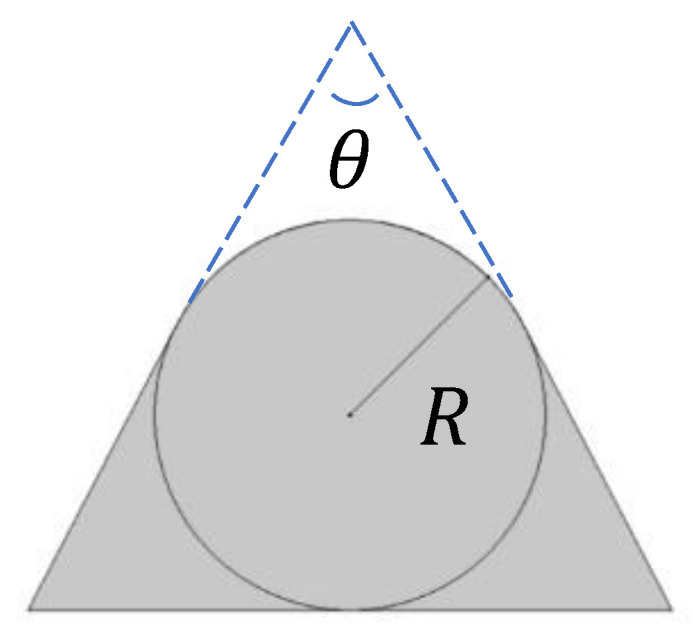
Schematic cross-section (in light grey) of the piston channel with a radius of curvature R at the bottom of the channel. *θ* is the opening angle.

The channel cross-section is depicted in Figure 3, wherein the opening angle of the channel is noted *θ* and the radius of curvature *R*. The flow of the incompressible medium is assumed to be laminar with negligible singular losses.

The fluidic resistance *β* of the channel per unit of length, according to the Darcy-Weisbach equation [37], is, in agreement with [29]:

(3) $$\beta =\frac{32\eta }{D_{h}^{2}.Area}=\frac{256\eta }{D_{h}^{4}\left(\pi -\theta +4\left(\tan\frac{\theta }{2}+\frac{1}{\cos\frac{\theta }{2}}\right)\right)}$$

where *D_h_* is the hydraulic diameter defined as:

(4) $$D_{h}=4\frac{Area}{Wetted\;perimeter}=2R$$

Several assumptions are made to estimate the position of the piston at different values of the applied pressure:

- *L_min_ = L_p_ – L*_1_ > 0 (i.e., there is no free flow at low pressure).
- the channel cross-section is regular.
- the spring stiffness *k* is constant (i.e., independent from the compression).
- Δ*L_S_*_0_ = *L_min_* (the condition for a constant flow rate).
- $R\;\gg D_{h}$ (the effective surface for pressure force calculation is not affected by the channel).
- *L_c_* = *L_reg_* (to simplify the numeral modeling).

The effective channel entrance is neglected if the hydraulic diameter is much smaller than the channel length. The effective channel length, considering the correction due to the geometry at the inlet and outlet of the channel, is, for $D_{h}\ll L_{ch}$:

(5) $$L_{ch\;eff}=L_{ch}-\frac{D_{h}}{\tan\left(\theta \right)}=L_{ch}-D_{h}\frac{L_{loop}}{pitch}=L_{ch}-\alpha D_{h}\approx L_{ch}$$

**Figure 4:**
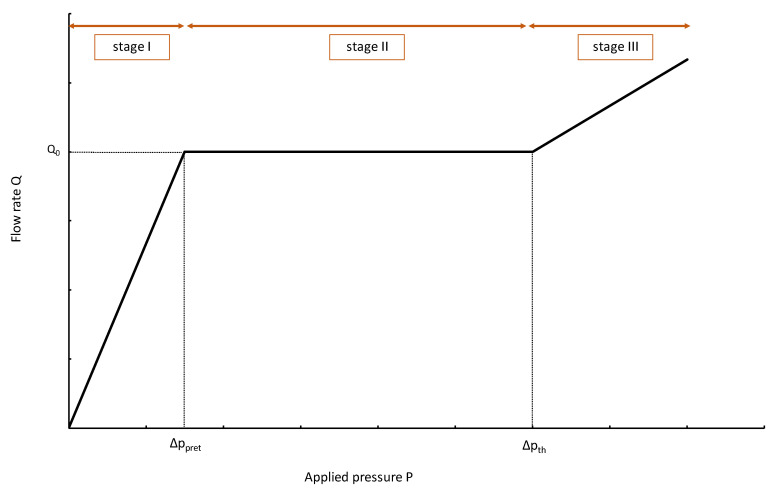
Ideal flow rate versus pressure gradient characteristic of a three-stage passive constant flow regulator. At low pressure (Stage I), the regulator has a constant and low hydraulic resistance. In Stage II, the flow is regulated at *Q*_0_ for a pressure gradient in the range [Δ*P_pret_*; Δ*P_th_*]. At high pressure (Stage III), the device delivers a large flow rate.

According to [1], the fluidic characteristic of a flow regulator can be split into three different stages, as illustrated in Figure 4:

- Stage I—low-pressure stage
- Stage II—flow-regulation stage
- Stage III—high pressure

##### Stage I

The minimum pressure (or pretension) *P_pret_* to move the piston is:

(6) $$P_{pret}=\frac{k\Delta L_{S0}}{S}=\frac{k\;L_{min}}{S}$$

If *P* < *P_pret_* and the outlet groove length *L*_2_ = 0, then the piston displacement Δ*L* is equal to zero and the fluidic resistance is constant:

(7) $$\{\begin{array}{c} \Delta L=0\\ Rf_{0}=\alpha \beta L_{min}=Cte \end{array}$$

Thus, the flow rate in Stage I varies linearly with the pressure:

(8) $$Q\left(P<P_{pret}\right)=\frac{P}{R_{f0}}=\frac{P}{\alpha \beta L_{min}}$$

and reaches, at *P* = *P_pret_*:

(9) $$Q\left(P=P_{pret}\right)=\frac{P_{pret}}{R_{f0}}=\frac{P_{pret}}{\alpha \beta L_{min}}=\frac{k}{\alpha \beta S}$$

##### Stage II

If *P_pret_* < *P* < *P_th_*, the piston displacement and the fluidic resistance become:

(10) $$\{\begin{array}{c} \Delta L=\frac{S\left(P-P_{pret}\right)}{k}\\ R_{f}=\alpha \beta \left(L_{min}+\Delta L\right)=\alpha \beta \left(L_{min}+\frac{S\left(P-P_{pret}\right)}{k}\right) \end{array}$$

The fluidic resistance varies with the applied pressure to compensate for the pressure variation. The flow rate *Q* in Stage II is therefore:

(11) $$Q\left(P_{pret}<P<P_{th}\right)=\frac{P}{\frac{P_{pret}}{Q\left(P=P_{pret}\right)}+\alpha \beta \frac{S\left(P-P_{pret}\right)}{k}}=\frac{P}{\frac{P_{pret}}{Q\left(P=P_{pret}\right)}+\frac{\left(P-P_{pret}\right)}{Q\left(P=P_{pret}\right)}}$$

which simplifies into:

(12) $$\left(P_{pret}<P<P_{th}\right)=Q\left(P=P_{pret}\right)=\frac{k}{\alpha \beta S}=Cte$$

According to the above hypothesis, the flow in Stage II is unaffected by pressure variations.

##### Stage III

At higher pressures, for *P* < *P_th_*, and considering that there are no openings in the cylinder:

(13) $$\{\begin{array}{c} \Delta L_{th}=\frac{S\left(P_{th}-P_{pret}\right)}{k}\\ R_{f}=\alpha \beta \left(L_{min}+\Delta L_{th}\right)=Cte \end{array}$$

The fluidic resistance is constant again and the flow rate increases with the applied pressure:

(14) $$Q\left(P>P_{th}\right)=\frac{P}{\alpha \beta \left(L_{min}+\Delta L_{th}\right)}$$

In the presence of cylinder openings of length *L*_2_ (see Figure 2), the part of the piston channel engaged into the narrowest part of the cylinder, after having reached a maximum, is reduced at high pressure. This part of the piston that is fluidically active is equal to:

(15) $$\Delta L+L_{min}\;if\;0<\Delta L<L_{reg}-L_{min}$$

Or

(16) $$L_{p}-\Delta L+L_{reg}-L_{min}\;if\;\Delta L\geq L_{reg}-L_{min}$$

Free flow conditions can be obtained at high pressure if the medium can bypass the channel and directly flow through to the openings of the cylinder that have very small fluidic restrictions.

#### 2.1.2. Non-Uniform Channel

If the channel is not uniform and if $L_{2}\neq 0$, special care should be taken to derive the fluidic resistance of the channel as a function of the pressure.

We consider for instance a piston wherein the hydraulic diameter is not constant along the helix: the initial part of the piston (length *L_min_*) that is engaged in the narrowest part of the cylinder is characterized by the coefficients *α*_1_ and *β*_1_ while the other part of the piston (length *L_p_* − *L_min_*) is characterized by *α*_2_ and *β*_2_.

If $0\leq \Delta L\leq L_{reg}-L_{min}$ and $L_{p}\geq L_{reg}$, the fluidic resistance of the channel is now:

(17) $$R_{f}=\alpha_{1}\beta_{1}L_{min}+\alpha_{2}\beta_{2}\Delta L=\alpha_{1}\beta_{1}L_{min}+\alpha_{2}\beta_{2}\frac{S\left(P-P_{pret}\right)}{k}$$

The flow rate in Stage II takes the form:

(18) $$Q=\frac{P}{\alpha_{1}\beta_{1}L_{min}+\alpha_{2}\beta_{2}\frac{S\left(P-P_{pret}\right)}{k}}=\frac{P}{\frac{P_{pret}}{Q\left(P=P_{pret}\right)}+\frac{\alpha_{2}\beta_{2}}{\alpha_{1}\beta_{1}}\times \frac{\left(P-P_{pret}\right)}{Q\left(P=P_{pret}\right)}}$$

Finally,

(19) $$\frac{Q}{Q\left(P=P_{pret}\right)}=\frac{P}{P_{pret}}\times \frac{1}{1+\frac{\alpha_{2}\beta_{2}}{\alpha_{1}\beta_{1}}\left(\frac{P}{P_{pret}}-1\right)}$$

For $\alpha_{1}\beta_{1}\neq \alpha_{2}\beta_{2}$ the flow rate profile is no longer flat as increases of the inlet pressure.

If $L_{reg}-L_{min}\leq \Delta L\leq L_{reg}$ and $L_{p}\geq L_{reg}+L_{min}$ the flow rate can be estimated using the following formulation of the fluidic resistance:

(20) $$R_{f}=\alpha_{1}\beta_{1}\left(L_{reg}-\Delta L\right)+\alpha_{2}\beta_{2}\Delta L=\alpha_{1}\beta_{1}L_{reg}-\Delta L\left(\alpha_{1}\beta_{1}-\alpha_{2}\beta_{2}\right)$$

These formulae are useful to obtain a specific regulation profile. Depending on the complexity of the target profile, modulation of the channel pitch or change in the channel cross-section can be considered.

#### 2.1.3. Gravity Effect and Device Orientation

The weight of the piston is an additional force that should be considered to refine the model. The angle between the horizontal axis and the piston axis is noted as φ. The effective weight of the piston (after correction due to Archimedes’ force) is noted as m. The minimum applied pressure to move the piston becomes:

(21) $$P_{pret}=\frac{k\;L_{min}-mg\sin\varphi }{S}$$

The flow rate at *P = P_pret_* is, therefore:

(22) $$Q\left(P=P_{pret}\right)=\frac{P_{pret}}{R_{f0}}=\frac{k\;L_{min}-mg\sin\varphi }{\alpha \beta SL_{min}}=\frac{k}{\alpha \beta S}-\frac{mg\sin\varphi }{\alpha \beta SL_{min}}$$

In Stage II, for *P_pret_* < *P* < *P_th_*, the piston displacement and the fluidic resistance are:

(23) $$\{\begin{array}{c} \Delta L=\frac{S\left(P-P_{pret}\right)}{k}\\ R_{f}=\alpha \beta \left(L_{min}+\Delta L\right)=\alpha \beta \left(L_{min}+\frac{S\left(P-P_{pret}\right)}{k}\right) \end{array}$$

The corresponding flow rate is:

(24) $$\begin{array}{cc} Q\left(P_{pret}<P<P_{th}\right) & =\frac{P}{\frac{P_{pret}}{Q\left(P=P_{pret}\right)}+\alpha \beta \frac{S\left(P-P_{pret}\right)}{k}}\\ & =\frac{P}{\frac{P_{pret}}{Q\left(P=P_{pret}\right)}+\frac{\left(P-P_{pret}\right)}{Q\left(P=P_{pret}\right)\left(1+\frac{mg\sin\varphi }{SP_{pret}}\right)}}\neq Cte \end{array}$$

The flow rate is not perfectly constant if the effective mass of the piston cannot be neglected.

#### 2.1.4. Out-of-Channel Flow

The difference in diameter between the cylinder and the piston induces a leakage between the loops of the channel. It is assumed that this parasitic flow is proportional to the active height of the piston equal to $\frac{L_{ch\;eff}}{\alpha }$ and normal to the channel flow. This leakage is considered effective outside of the channel over a distance equal to:

(25) $$\frac{L_{ch\;eff}}{\alpha }\times \left(1-\frac{w_{ch}}{2\pi b}\right)=\frac{L_{ch\;eff}}{L_{loop}}\times \left(2\pi b-w_{ch}\right)$$

where *w_ch_* is the channel width. In a first approximation, the piston and the cylinder are concentric and thus the fluidic resistance of the parasitic flow per unit of length is [38]:

(26) $$R_{f\;losses}/L=\frac{8\eta }{\pi \left(R_{c}^{4}-R^{4}-\frac{{\left(R_{c}^{2}-R^{2}\right)}^{2}}{ln\frac{R_{c}}{R}}\right)}$$

The total parasitic fluidic resistance is parallel to the channel flow is, therefore:

(27) $$R_{f\;losses}=\frac{8\eta }{\pi \left(R_{c}^{4}-R^{4}-\frac{{\left(R_{c}^{2}-R^{2}\right)}^{2}}{ln\frac{R_{c}}{R}}\right)}\times \frac{L_{ch\;eff}}{L_{loop}}\times \left(2\pi b-w_{ch}\right)$$

The combination of both resistances in parallel leads to the global fluidic resistance equal to:

(28) $$R_{f}=\frac{R_{f\;ch}\times R_{f\;losses}}{R_{f\;ch}+R_{f\;losses}}$$

where $R_{f\;ch}$ is the fluidic resistance of the channel.

#### 2.1.5. Other Fluid Restrictions

The fluidic line exhibits a fluidic resistance $R_{f\;line}$. If this resistance is not negligible, this additional pressure drop shall be considered. For a given pressure gradient in the valve $\Delta P_{valve}$, the flow rate *Q* is calculated according to the model described hereabove.

The additional pressure drop $\Delta P_{line}$ due to $R_{f\;line}$ is:

(29) $$\Delta P_{line}=Rf_{line}Q$$

The effective pressure gradient that generates the flow rate *Q* is:

(30) $$\Delta P_{eff}=\Delta P_{valve}+\Delta P_{line}$$

It is noteworthy that the resulting curve may exhibit points that cannot be obtained experimentally (see for instance the curve obtained at 60 mm (Sim60) in Figure 10 as well as the discussion about hysteresis).

An alternative method consists of directly solving the flow equation.

It is first assumed that the pressure gradient along the channel is larger than $P_{pret}$.

Thus,

(31) $$\Delta P_{eff}=\Delta P_{valve}+\Delta P_{line}=\left(R_{f0}+R_{f\;line}+\gamma \left(\Delta P_{eff}-R_{f\;line}Q-P_{pret}\right)\right)Q$$

where $\gamma =\frac{\alpha \beta S}{k}$.

The resulting flow equation is:

(32) $$\gamma R_{f\;line}Q^{2}-\left(R_{f0}+R_{f\;line}+\gamma \left(\Delta P_{eff}-P_{pret}\right)\right)Q+P=0$$

With general solutions of the form:

(33) $$Q=\frac{\varphi \pm \sqrt{\varphi^{2}-4\gamma R_{f\;line}\Delta P_{eff}}}{2\gamma R_{f\;line}}$$

with

(34) $$\varphi =R_{f0}+R_{f\;line}+\gamma \left(\Delta P_{eff}-P_{pret}\right)$$

The first hypothesis shall be verified using the estimated value of the flow rate.

- If $\Delta P_{eff}-R_{f\;line}Q-P_{pret}\leq 0$, then the flow is simply given by:

(35) $$\Delta P_{eff}=\Delta P_{valve}+\Delta P_{line}=\left(R_{f0}+R_{f\;line}\right)Q$$

- If $\Delta P_{eff}-R_{f\;line}Q-P_{pret}>0$, both solutions of Equation (33) are possible depending on the initial conditions (see the discussion about hysteresis).

### 2.2. Simulation

The model was coded and simulated using a visual programming language (LabVIEW, National Instruments). Each design parameter was introduced as a controller. Out-of-channel flow (leakage) is also considered as an option. The piston displacement and the flow rate are computed in the predefined inlet pressure range using constant pressure steps.

### 2.3. Prototyping

**Figure 5:**
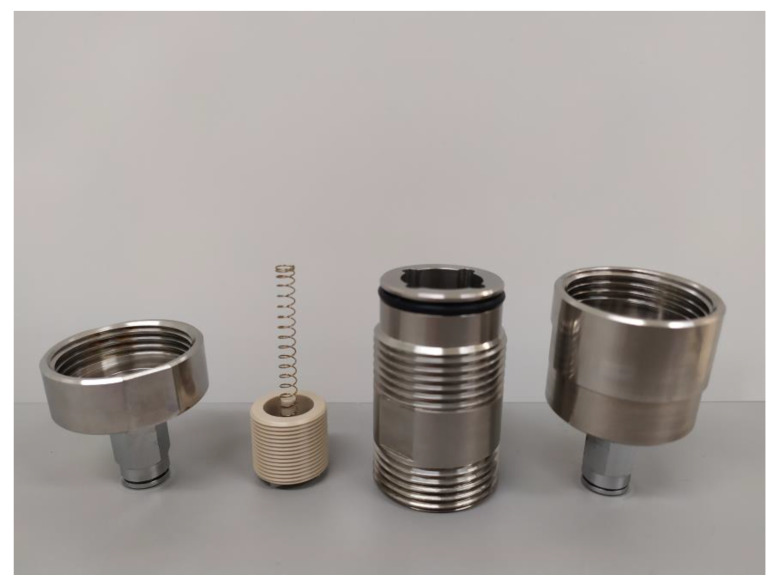
Prototype teardown showing, from left to right: the inlet flange, the piston, and the spring, the cylinder, and the outlet flange. Nominal dimensions are provided in the mechanical drawings. The height of the piston made of PEEK is 2 mm shorter than expected and the clearance between the piston and the cylinder is larger than expected (inner diameter of the cylinder at 18.035 mm instead of 18.015 mm); the other parts are within specification.

**Figure 6:**
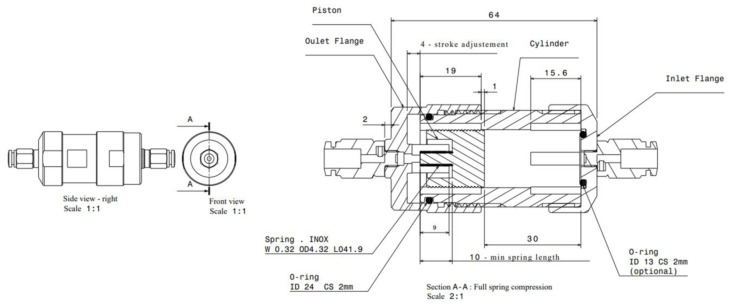
Side and front views of the device. The nominal dimensions are indicated in Section A-A.

**Figure A1:**
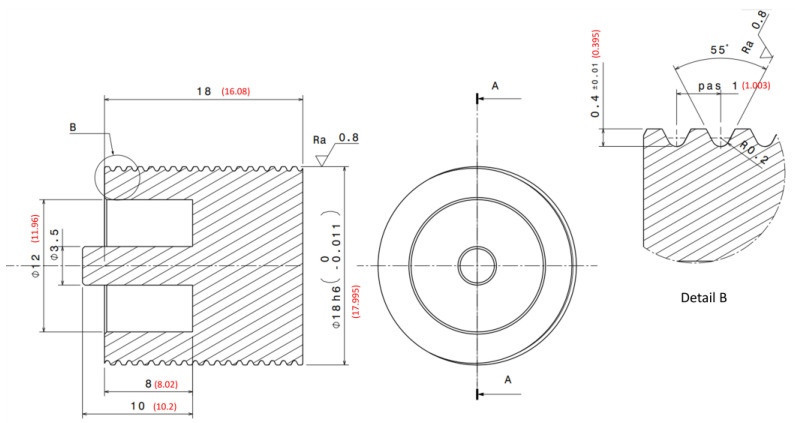
Mechanical drawing of the piston. Parentheses (red) indicate the measured dimensions of the sample.

**Figure A2:**
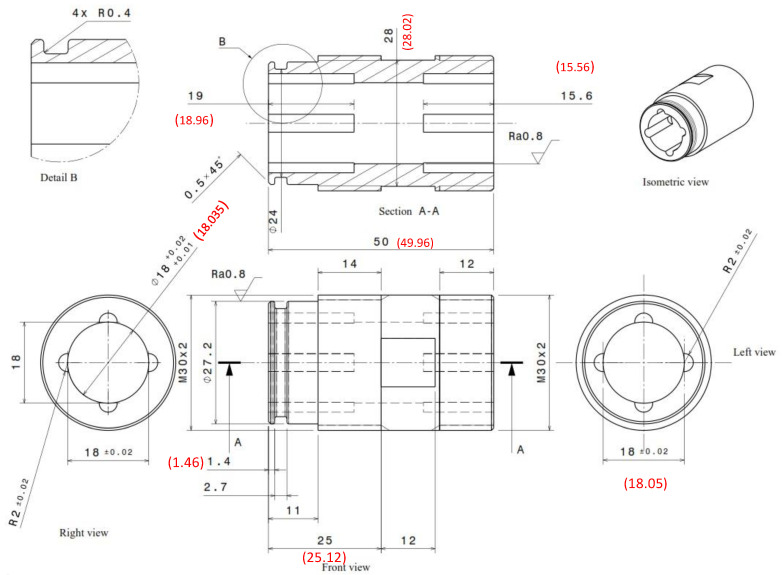
Mechanical drawing of the cylinder. Parentheses (red) indicate the measured dimensions of the sample.

**Figure A3:**
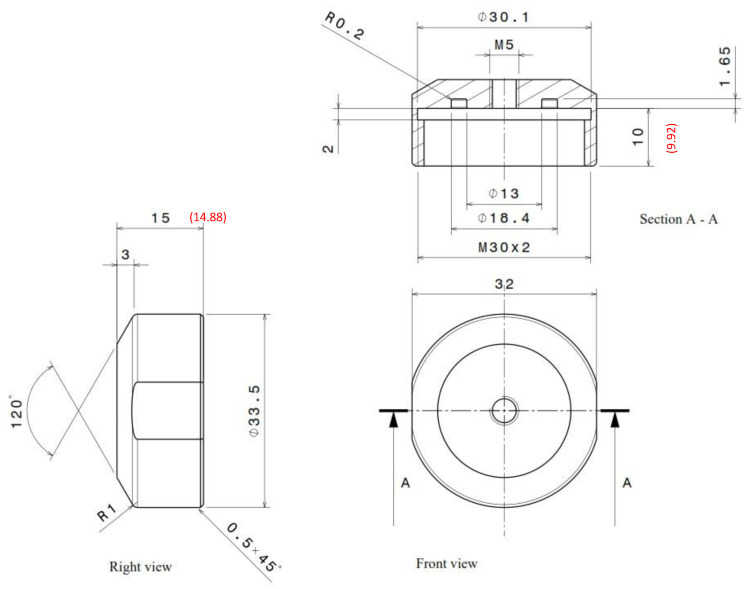
Mechanical drawing of the inlet flange. Parentheses (red) indicate the measured dimensions of the sample.

**Figure A4:**
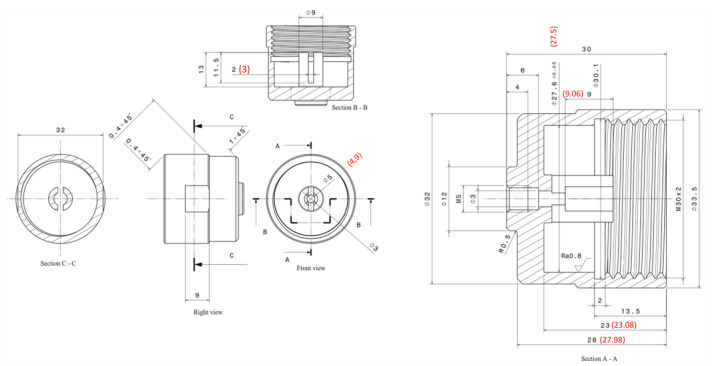
Mechanical drawing of the inlet flange. Parentheses (red) indicate the measured dimensions of the sample.

The prototype in stainless steel and PEEK (piston) was machined as illustrated in Figure 5, which shows the different elements of the passive adjustable flow regulator, respectively, the inlet flange, the piston and its spring, the cylinder, and the outlet flange. The assembly drawing is shown in Figure 6. The mechanical drawings of the sub-elements are provided in Appendix A (Figure A1, Figure A2, Figure A3 and Figure A4). The choice of materials and the external dimensions of the device were selected to obtain a prototype that is easy to machine and compatible with assembly and disassembly. The data obtained from metrology is indicated in parentheses. These values were used as input data for the numerical simulations. Since the piston is 2 mm shorter than expected, a 2 mm thick elastomeric disk was glue on the top of the cylinder to obtain the targeted spring preload and therefore limit the impact of this out-of-specs dimension onto the fluidic characteristic. Additionally, the clearance between the piston and the cylinder is larger than expected: the inner diameter of the cylinder is 18.035 mm instead of 18.015 mm. The spring dimensions and specifications are listed in Appendix B.

### 2.4. Adjusting the Flow Rate

**Figure 7:**
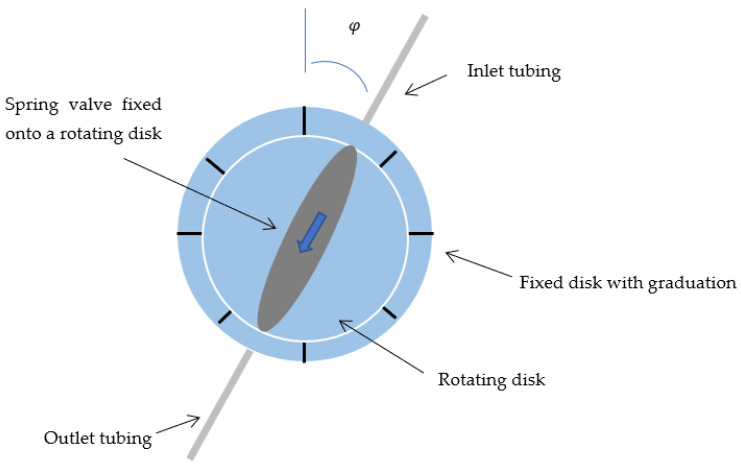
Spring valve mounted onto a rotation disk for gravimetric flow adjustment. The blue arrow indicates the direction of the flow. The rotation of the disk modifies the initial spring preload by gravimetry.

Several options can be considered to adjust the flow manually. The spring preload can be modified using the own weight *m* of the piston as illustrated in Figure 7.

For a rotation angle φ, the spring preload is equal to mgcosφ, where $g=9.81\;{m\;s}^{-2}$ is the gravitational acceleration. As illustrated in Figure 7, the predefined position of the rotating disk allows adjustment of the flow by gravimetry, the vertical position with the outlet above in the inlet corresponding to the higher flow rate.

**Figure 8:**
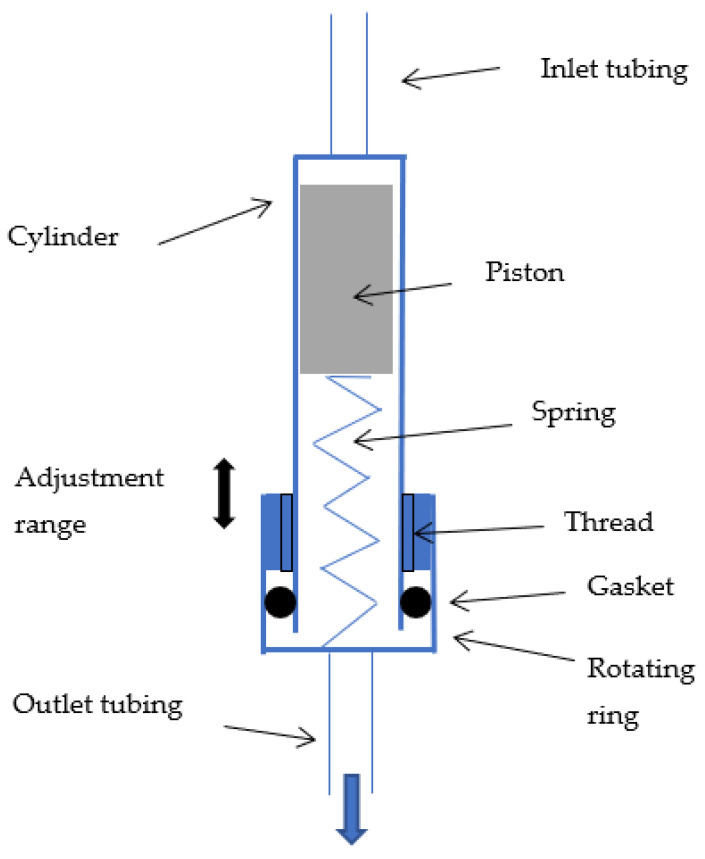
Adjustable flow-control spring valve with a rotating ring that allows the spring preload to be changed manually. A gasket between the ring and the cylinder is used to prevent leakage.

The simplest method that was considered in this study consists of changing the spring preload with a rotating ring as illustrated schematically in Figure 8. The user turns the ring to modify the initial spring compression and therefore the fluidic characteristic of the device. Three different Positions 0, 1, and 2 were considered here, corresponding, respectively, to flange-to-flange distances of 64, 62, and 60 mm (see Figure 6 for Position 0 at 64 mm).

A more complex design includes a second colinear compression spring that becomes active either in a passive way at a given threshold pressure to increase the flow rate at high pressure, or actively by manual activation using a sliding sleeve that engages the second spring against the movable piston. According to [29], the valve at high flow could exhibit a free flow and become occlusive depending on the application and the need for a purge mode.

### 2.5. Adjusting the Pressure-Regulation Range

#### 2.5.1. Regulation Threshold at Low Pressure

A rotating ring similar to the one described in Figure 8 is placed at the inlet of the device to adjust, using a shaft, the initial position of the piston and therefore the minimum value of the pressure-regulation range. As long as the spring is under compression, the shaft motion through the ring rotation induces a displacement of the piston.

#### 2.5.2. Regulation Threshold at High Pressure

The maximum value of the pressure-regulation range is tuned through another shaft attached to the center of the ring. This shaft acts as a mechanical stop for the piston at high pressure. Rotating the ring induces a change in the spring maximum compression and thus a modification of the high-pressure part of the regulation range. The shaft should not be attached to the spring support to avoid any modification of the spring preload.

The different setting methods can of course be combined altogether in a single device. In this case, the shaft used to change the position of the mechanical stop limiting the motion of the piston at high pressure is mounted on the spring support ring through an additional ring.

### 2.6. Fluidic Characterization

**Figure 9:**
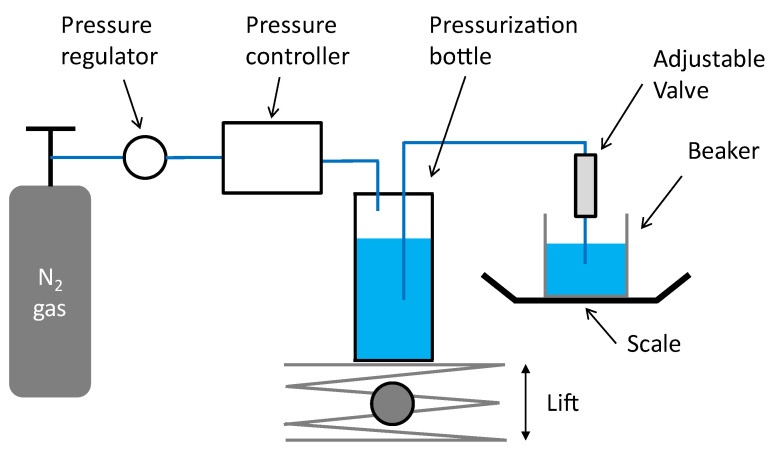
Schematic representation of the test setup.

Fluidic characterizations have been performed using water ISO 3 as the test medium. The test setup is shown in Figure 9.

The water is introduced into a pressurization bottle connected to a Druck DPI520 pressure controller. The fluid is forced to flow through the valve and is collected into a beaker placed onto a Sartorius MC1 LP620P scale. Pressure steps of 5 and 10 mbar have been used at low pressure. The typical stabilization time is 1 minute. The flow rate is estimated from weight measurements across 30 s, using a density of 1 g/mL at room temperature (20 °C). The valve is placed in the vertical position and tested using positive and negative steps of pressure.

As shown in Figure 9, special care has been taken to limit the water column of the fluidic line to less than 5 cm throughout the test. Three different settings of the valve have been tested, corresponding to a relative displacement of the outlet flange of 0 mm (Position 0), 2 mm (Position 1), and 4mm (Position 2) from its nominal Position. The setting is controlled by a caliper that is used to measure the distance from the inlet to outlet flanges of, respectively, 64 mm, 62 mm, and 60 mm (see Figure 6).

## 3. Results

### Experimental Results

**Figure 10:**
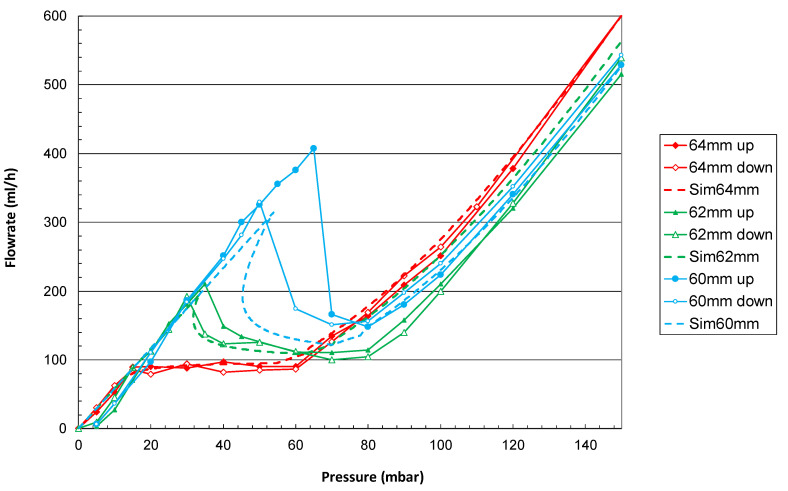
Flow rate versus pressure characteristics of the valve for the three different settings 0, 1, and 2 (corresponding to a flange-to-flange distance of, respectively, 64, 62, and 60 mm) using positive (up) and negative (down) pressure steps. The valve is tested vertically, with the inlet above the outlet. Dashed lines represent the numerical simulations of the valve for each setting.

The flow characteristics of the device at Positions 0 (64 mm), 1 (62 mm), and 2 (60 mm) are presented in Figure 10 and compared to simulations that consider the data obtained during the prototype metrology, out-of-channel flow, and the fluidic resistance of the fluidic line as well. The pressure-regulation range matches the theoretical expectations, considering a fluidic line resistance estimated experimentally to be about $4.3\times 10^{10}\;\mathrm{Pa}\;s/m^{3}$.

Table 1 provides the pressure-regulation ranges and the mean flow rate for each setting. As expected, the increase of the spring preload by reducing the piston stroke induces an increase in the mean flow rate, since the piston requires more pressure force to initiate its movement. The match between the simulation and experiment is good at Positions 0 and 2. The fitting of the experimental data is more difficult at Position 3 because 3 hysteresis becomes important, and the regulation range is very narrow. In addition, simulated curves show a crossover point that cannot be measured experimentally, as discussed in Section 2.1.5.

## 4. Discussion

### 4.1. Flow Regulation and Variability

The prototype was dimensioned to exhibit the nominal constant flow rate at the minimum spring preload (Position 0). As any change of the spring preload induces both a slope of the flow versus pressure characteristics together with a lowering of pressure-regulation range (see Figure 10 and Section 2.1), the design of a “flat” characteristic at an intermediate position is recommended, in order to explore both the positive and negative changes in the spring preload. Therefore, it is possible to obtain a flat profile across a wider range of settings. These results show that under constant temperature conditions, the flow rate can be adjusted up to at least +/−30% by a simple rotation of the outlet flange by a few mm.

To maximize the channel cross-section and thus the tolerance to contamination, the prototype piston was designed with a very small spiral channel pitch. However, this also results in a high sensitivity to leakage out of the channel. Thus, the manufacturing capability must be considered when redesigning the system to limit the impact of machining tolerances on flow-rate variability. A compromise must be found between increasing the pitch and reducing the cross-sectional area of the channel and/or the piston diameter, which has an impact on sensitivity to contamination. An example of a design that has less sensitivity to manufacturing tolerances is provided in Section 4.3. Overall, flow regulation was achieved using a hydraulic diameter that is at least one order of magnitude larger than MEMS-based devices and other flow-controlled shunts [1,13,23]. Thus, this technology addresses one of the main concerns raised about using a flow-controlled valve for CSF drainage. The ability to flush the valve is also an attractive feature because the working fluid may be hemorrhagic. Finally, the adjustability enables the use of a single device for all patients with EVD and allows for a simplified weaning procedure.

### 4.2. Hysteresis

Hysteresis is intrinsically linked to the valve design and the additional fluidic resistance of the line. The flow characteristic obtained in Position 2 exhibits two different flow rates for a given pressure gradient within the range (45; 55) mbar. This phenomenon is due to the additional fluidic resistance of the line. As the applied pressure increases, the flow rate increases linearly as long as the piston remains in its initial position (no movement). Pressure drops are observed along the channel of the piston and the fluidic line itself. When the pressure gradient onto the piston surface is large enough to overcome the spring pretension, the piston moves to an equilibrium position.

At this given applied pressure *P*_1_, the fluidic resistance of the channel $R_{f\;ch}$ will increase as well as the whole resistance of the device equal to $R_{f\;ch}+R_{f\;line}$.

This results in a decrease in the flow rate *Q*

(36) $$Q=\frac{P_{1}}{R_{f\;ch}+R_{f\;line}}$$

A lower flow rate requires a lower pressure at the inlet of the fluidic line with a constant fluidic resistance $R_{f\;line}.$.

As the pressure reaches *P*_1_ (the minimal applied pressure necessary to move the piston from rest), any increase in applied pressure will induce a lowering of the pressure downstream of the piston according to the mechanism described previously. This pressure lowering will induce an additional movement of the piston and progressively the piston will move until the system reaches an equilibrium position satisfying the flow equation described in Equation (32).

Without inlet pressure fluctuations, this forward movement of the piston begins at pressure:

(37) $$P_{forward\;mov.}=P_{pret}\left(1+\frac{R_{f\;line}}{R_{f0}}\right)$$

Conversely, when the piston is at a full stroke (high pressure) and considering negative pressure steps, the piston starts the backward movement at a pressure equal to:

(38) $$P_{backward\;mov.}=P_{th}\left(1+\frac{R_{f\;line}}{R_{f1}}\right)$$

where $P_{th}$ is equal to the pressure gradient onto the piston that can push it against the stop limiter, $R_{f1}$ being the fluidic resistance of the channel in this later position.

We infer that this hysteresis is notably a function of the parameters $R_{f\;line}$, $R_{f0}$, and $R_{f1}$.

This effect can be used to create an adjustable, automatic shut-off valve that prevents overflow in case of a sudden increase in the flow rate. This valve could be reversible or not (the piston can be blocked mechanically in a high position). On the other hand, hysteresis can be limited by limiting the pressure drops along the fluidic line.

### 4.3. Application to CSF Drainage

As the fluidic model shows a pretty good match with the experiment, new simulations were performed to adapt the current design to the flow and pressure range requirements of the CSF drainage.

**Figure 11:**
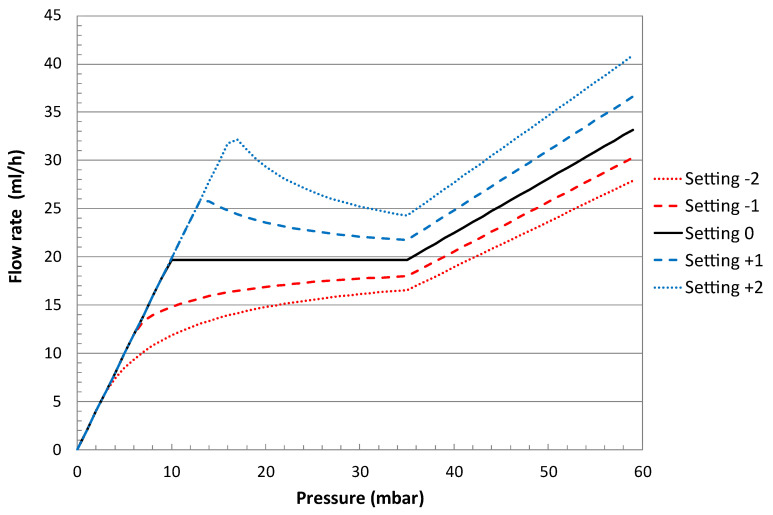
Simulated fluidic characteristics of a device described in [29] at five different settings corresponding to adjustment of the cylinder length from 13.5 mm to 15.5 mm using steps of 0.5 mm. The fluid medium is water at 37 °C.

The nominal flow rate was set at 20 mL/h at 37 °C, considering that CSF is equivalent to water in terms of viscosity [29]. The pressure-regulation range for an EVD can be set at 10 to 35 mbar or more. The design proposed in [29] for CSF drainage was chosen as a reference. The flow adjustment is obtained by changing the effective cylinder length with the rotating flange from 13.5 mm to 15.5 mm using steps of 0.5 mm, the nominal value being 14.5 mm. The simulated fluidic characteristics are shown in Figure 11, for a device in a horizontal position.

**Figure 12:**
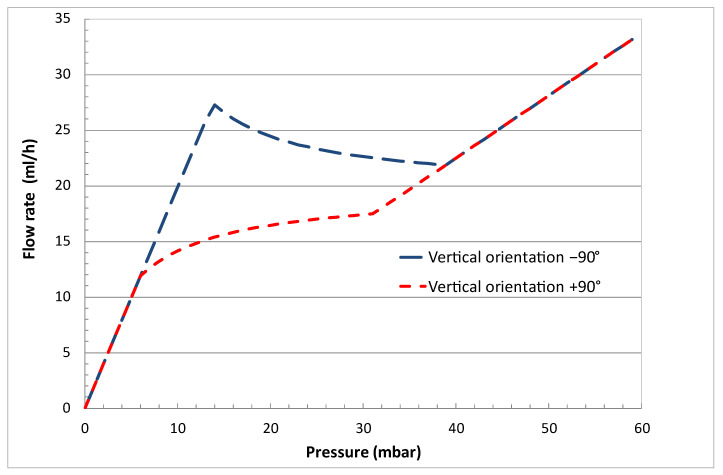
Simulated fluidic characteristics of a device described in [29] at two different vertical orientations (+90° with the inlet above the outlet and −90° with an outlet above the inlet). The fluid medium is water at 37 °C.

An interesting alternative method consists of using the piston weight and the orientation of the device to change the fluidic characteristic of the device. Figure 12 shows the simulation of a device described in [29], for two different orientations of the vertical piston (respectively, +90° with the inlet above the outlet and −90° with the outlet above the inlet), the effective weight of the piston here being equal to 500 mg. The device can be simply mounted onto a rotating disk with predefined positions, each position corresponding to a predetermined fluidic characteristic. The two methods can be combined to extend the range of accessible flow rates.

## 5. Conclusions

The nominal flow rate of a constant flow regulator is commonly set at the time of manufacture. However, an adjustment of this value by the end user could be desirable in several applications, including CSF drainage. Therefore, the adjustment of a flow regulator with a spring valve design was investigated here. A fluidic model was built, and a prototype was designed and tested at a constant temperature. The results show that the flow rate can be adjusted up to at least +/−30% from the nominal value by turning a flange a few mm. A numerical model was used to refine the design for CSF drainage application, specifically external ventricular drainage. This simple model offers the possibility to easily adjust a flow-regulated valve without the need for complex 3D FEM simulation tools with fluid-structure interactions. This feasibility study, which is a first attempt to realize the adjustability of a flow-regulated valve, opens new perspectives in the field of EVD and in any other application where the target flow rate is not known in advance. The next steps include fabrication and testing of devices dedicated to EVD, including evaluation of sensitivity to contaminants, self-cleaning and purge-mode capabilities, as well as usability testing for manual adjustment.

## Figures and Tables

**Table 1:** Regulation range and mean flow rate of the device at the three different settings.

	Position 0 (64 mm)		Position 1 (62 mm)		Position 2 (60 mm)	
	Experiment	Simulation	Experiment	Simulation	Experiment	Simulation
Regulation range (mbar)	[+15; +60]	[+15; +60]	[+30; +80]	[+40; +80]	[+60; +80]	[+52; +78]
Mean flow rate mL/h	91	92	120	114	160	130
St. dev. (%)	4	4	14	4	12	5.5

## Data Availability

Not applicable.

## Nomenclature

**Symbol**	**Name**
$L_{c}$	Cylinder length
$L_{p}$	Piston length
$L_{1}$	Inlet groove length
$L_{2}$	Outlet groove length
$L_{reg}$	Regulation length, with $L_{c}=L_{1}+L_{reg}+L_{2}$
*k*	Spring stiffness
$D_{h}$	Hydraulic diameter of the channel
2πb	Helix pitch
$L_{loop}$	Length of one loop of the helicoidal channel
R	Piston radius
$R_{c}$	Cylinder radius
θ	Channel angle
η	Dynamic viscosity
$L_{ch}$	Active channel length
P	Applied pressure
S	Piston active surface
$L_{min}$	Minimum length of the piston engaged into the narrowest part of the cylinder
$R_{f}$	Fluidic resistance
$R_{f\;ch}$	Fluidic resistance of the channel
$R_{f\;losses}$	Parasitic fluidic resistance between the cylinder and piston
$R_{f\;line}$	Fluidic resistance of the fluidic line
ΔL	Piston displacement
$P_{pret}$	Minimum pressure to move the piston (pretension)
$P_{th}$	Minimum pressure to reach the piston mechanical stop
$L_{S\;free}$	Free length of the spring
$L_{S\;min}$	Minimum spring length
$\Delta L_{S0}$	Initial spring compression length $=\;L_{S\;free}-\left(L_{c}-L_{p}\right)$
$L_{h}$	Piston recess
*α*	Channel length per unit of piston length $=L_{loop}/2\pi b$
*β*	Fluidic resistance of the channel per unit of channel length
φ	Device rotation angle
*m*	Piston effective weight
*g*	Gravitational acceleration
**Abbreviation**	**Name**
CSF	Cerebrospinal fluid
EVD	External Ventricular Drain
ICP	IntraCranial Pressure
MEMS	Microelectromechanical system
NPH	Normal-pressure hydrocephalus
PDMS	Poly(dimethylsiloxane)
PEEK	Polyether ether ketone

## Appendix B. Spring Dimensions and Specifications

Material	Stainless Steel
Wire diameter	0.32 mm
External diameter	4.32 mm
Free length $L_{S\;free}$	41.9 mm
Min spring length *L_min_*	10 mm
Max loaded length	9.68 mm
Solid length	6.9 mm
Spring stiffness *k*	0.0776 N/mm
